# Stress-induced endocytosis and degradation of epidermal growth factor receptor are two independent processes

**DOI:** 10.1186/s12935-016-0301-x

**Published:** 2016-03-31

**Authors:** Ke Peng, Qian Dai, Jing Wei, Genbao Shao, Aiqin Sun, Wannian Yang, Qiong Lin

**Affiliations:** School of Medicine, Jiangsu University, 301 Xuefu Road, Zhenjiang, China

**Keywords:** Caspase, Degradation, EGFR, Endocytosis, p38, Stress

## Abstract

**Background:**

Epidermal growth factor receptor (EGFR) is an important oncogenic protein in multiple types of cancer. Endocytosis and degradation of epidermal growth factor receptor (EGFR) are two key steps for down-regulation of cell surface level of EGFR and modulation of EGFR signaling. Stress conditions induce ligand-independent endocytosis and degradation of EGFR. However, it is not clear whether stress-induced endocytosis and degradation are consequential or two independent events.

**Methods:**

Endocytosis and degradation of EGFR in response to stress treatment and effects of the p38 inhibitor, the Caspase-3 inhibitor and the proteasomal inhibitor in cervical cancer HeLa cells were determined using immunoblotting and immunofluorescent staining assays.

**Results:**

Stress conditions, such as protein biosynthesis inhibition, UV light irradiation, and hyper-osmosis, induced both ligand-independent endocytosis and degradation of EGFR. Stress-induced endocytosis of EGFR relies on p38 kinase activity, while stress-induced degradation of EGFR is catalyzed by Caspase-3 activity. Inhibiting p38 kinase impairs only the endocytosis but not the degradation, while inhibiting Caspase-3 results in the opposite effect to inhibiting p38. Furthermore, proteasomal activity is required for stress-induced degradation of EGFR and cell death, but not for endocytosis.

**Conclusions:**

The results indicate that stress-induced endocytosis and degradation are two independent events and suggest stress signaling may utilize a double-secure mechanism to down-regulate cell surface EGFR in cancer cells.

## Background

The receptor tyrosine kinase (RTK) EGFR plays important roles in cell growth, survival, differentiation, and transformation [1–5]. Down-regulation of cell surface EGFR is a major process for attenuating EGFR signaling, especially for the signaling that occurs on plasma membrane [6–9]. Defects in down-regulation, mainly endocytosis and degradation processes, of cell surface EGFR may cause constitutive activation of cell proliferation and survival signaling and lead to oncogenesis and tumor progression [10–12].

There are two major pathways for down-regulation of cell surface EGFR in cells. One is the ligand-induced endocytosis and degradation of EGFR and the other is stress-induced endocytosis and degradation. The ligand-induced endocytosis and degradation of EGFR have been extensively studied [13–16]. Upon activation by a ligand, such as EGF, EGFR is tyrosine-phosphorylated, and subsequently recruits Cbl, an E3 ubiquitin ligase, and Grb2, an adaptor protein, for assembly of the ubiquitination complex, and interacts with Eps15 and AP-2, two endocytic adaptor proteins, to form clathrin-coated endocytic vesicles [17–19]. The endocytic vesicles or endosomes containing ubiquitinated EGFR are recognized by the ubiquitin-binding protein Hrs and transported to multi-vesicular bodies (MVBs) [20, 21]. Finally, the MVBs fuse with lysosomes to complete degradation of EGFR. In this ligand-induced endocytosis and degradation of EGFR, tyrosine phosphorylation and ubiquitination are two key biochemical processes, in which the tyrosine phosphorylation is required for organization of the endocytosis and ubiquitination complexes, and the ubiquitination functions as a sorting signal for transport of the EGFR-loaded vesicles to lysosomes. In addition, endocytosis and degradation are two consequential events in ligand-induced down-regulation of EGFR, in which the degradation process is dependent on the endocytosis.

Stress conditions, such as UV irradiation, inflammatory cytokines, osmotic stress, oxidation stress and protein biosynthesis inhibition, induce endocytosis and degradation of EGFR independent of ligand stimulation [22–24]. The stress-induced endocytosis and degradation of EGFR have distinguished biochemical processes from ligand-induced endocytosis and degradation. First, no tyrosine phosphorylation or ubiquitination is required for initiation of stress-induced endocytosis of EGFR [22]. Second, activation of p38 kinase is essential for stress-induced endocytosis of EGFR [22]. Third, lysosomes, which are required for ligand-induced degradation of EGFR, are not involved in stress-induced degradation [25]. However, assembly of endocytic vesicles in stress-induced endocytosis of EGFR, similar to in ligand-induced endocytosis, is mediated by the adaptor protein AP2 and coated by clathrin [26–28]. Whether stress-induced degradation of EGFR is dependent on proteasomes or caspases is still in dispute [22, 25]. Furthermore, the connection between endocytosis and degradation is not characterized in stress-induced down-regulation of EGFR.

Both ligand- and stress-induced endocytosis and degradation of EGFR have been observed in cancer cells [27, 29–31]. Most of studies on the ligand-induced endocytosis and degradation of EGFR have been characterized in cervical cancer HeLa cells. Although ligand-induced endocytosis was initially thought to be an important means to down-regulate cell surface EGFR, subsequent studies found that endocytic EGFR in cancer cells continues generating signals on endosomes that differ from on plasma membrane [32, 33]. The cellular signaling involving stress-induced endocytosis currently is not well understood and poorly characterized. It has been observed that stress-induced endocytosis potentiates the chemotherapy drug-induced apoptosis [27, 29]. A very recent report found that in HeLa cells UV irradiation or cisplatin-induced endocytosis of EGFR utilizes a completely different trafficking route from EGF-induced endocytosis [29]. The stress-induced endocytic EGFR is accumulated in a subpopulation of lyso-bisphosphatidic acid (LBPA)-rich multivescular bodies (MVBs) that are distinguishable from the MVBs containing EGF-induced endocytic EGFR [29]. Furthermore, impairment of the stress-induced EGFR endocytosis enhanced UV-irradiation or cisplatin-induced apoptosis [29], suggesting that the stress-induced endocytosis of EGFR might play a role in antagonizing the stress-induced apoptosis in cancer cells.

In this report, we show that stress conditions, including protein synthesis inhibition, UV irradiation, and hyperosmosis, cause endocytosis and degradation of EGFR in cervical cancer HeLa cells. Our data demonstrate that the stress-induced activation of p38 kinase is required for the endocytosis of EGFR while the activation of Caspase-3 is for the degradation. Furthermore, the p38 activation-induced internalization and the Caspase 3-mediated degradation are two independent events in stress-induced down-regulation of cell surface EGFR. However, these two processes seem coordinated in timing because the internalization precedes the degradation. We propose that stress conditions down-regulate cell surface EGFR in cancer cells by the two coordinated but independent processes, i.e., the p38-mediated endocytosis and the Caspase-3-catalyzed degradation of EGFR, to impede cancer cell proliferation.

## Results

### Protein synthesis inhibition, UV irradiation or hyperosmosis induces degradation of EGFR in HeLa cells

**Fig. 1:**
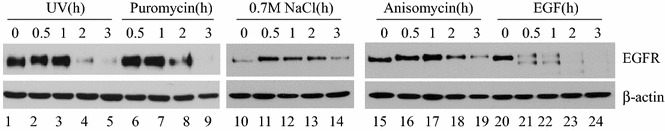
Both physical and chemical stress conditions induce degradation of EGFR. HeLa cells were cultured in DMEM plus 10 % FBS to 90 % confluence and serum-starved for 12 h. For puromycin (5 μg/ml), anisomycin (60 μM) or EGF (100 ng/ml) treatment, the stock solution of the chemical was added to the medium for indicated time. For 0.7 M NaCl treatment, the cells were cultured in serum-free DMEM medium plus 0.7 M NaCl for indicated time. For UV treatment, the cells were irradiated under a UV light (about 8 J/cm^2^) for 2 min, then cultured in serum-free medium for indicated time. The cells were lysed and EGFR was immunoprecipitated with anti-EGFR (Mab528) and detected by immunoblotting with anti-EGFR(1005). The amount of lysates used for Immunoprecipitation was controlled by immunoblotting of β-actin (bottom panels) in whole cell lysates with anti-β-actin

Previous studies have shown that treatments of anisomycin or UV irradiation with cervical cancer HeLa cells induced degradation of EGFR [22]. To confirm the effect of stress conditions on EGFR endocytosis and degradation, in addition to UV irradiation and anisomycin, we also examined effects of 0.7 M NaCl (hyper-osmosis) and protein synthesis inhibitor puromycin on EGFR degradation in HeLa cells. As shown in Fig. 1, all the stress conditions caused significant degradation of EGFR upon 3 h treatments (lanes 5, 9, 14 and 19). In general, about 70–80 % of total EGFR in whole cell lysates was degraded upon 3 h treatment of stress conditions in HeLa cells (data not shown). Comparing to ligand (EGF)-induced degradation of EGFR (lanes 20–24), stress-induced degradation of EGFR has two distinguishable traits: (i) the degradation rate is much slower than that of ligand-induced degradation; and (ii) the degradation products are different from that of ligand-induced degradation. No degradation products were detected by immunoblotting with anti-EGFR (Santa Cruz, 1005) in stress treated cells, while a degraded EGFR band was detected upon 0.5, 1 and 2 h EGF stimulation (lanes 21–23, Fig. 1). These data suggest that the molecular mechanism underlying stress-induced degradation is different from that of ligand-induced degradation.

### Caspase activity but not p38 kinase activity is required for stress-induced degradation of EGFR

**Fig. 2:**
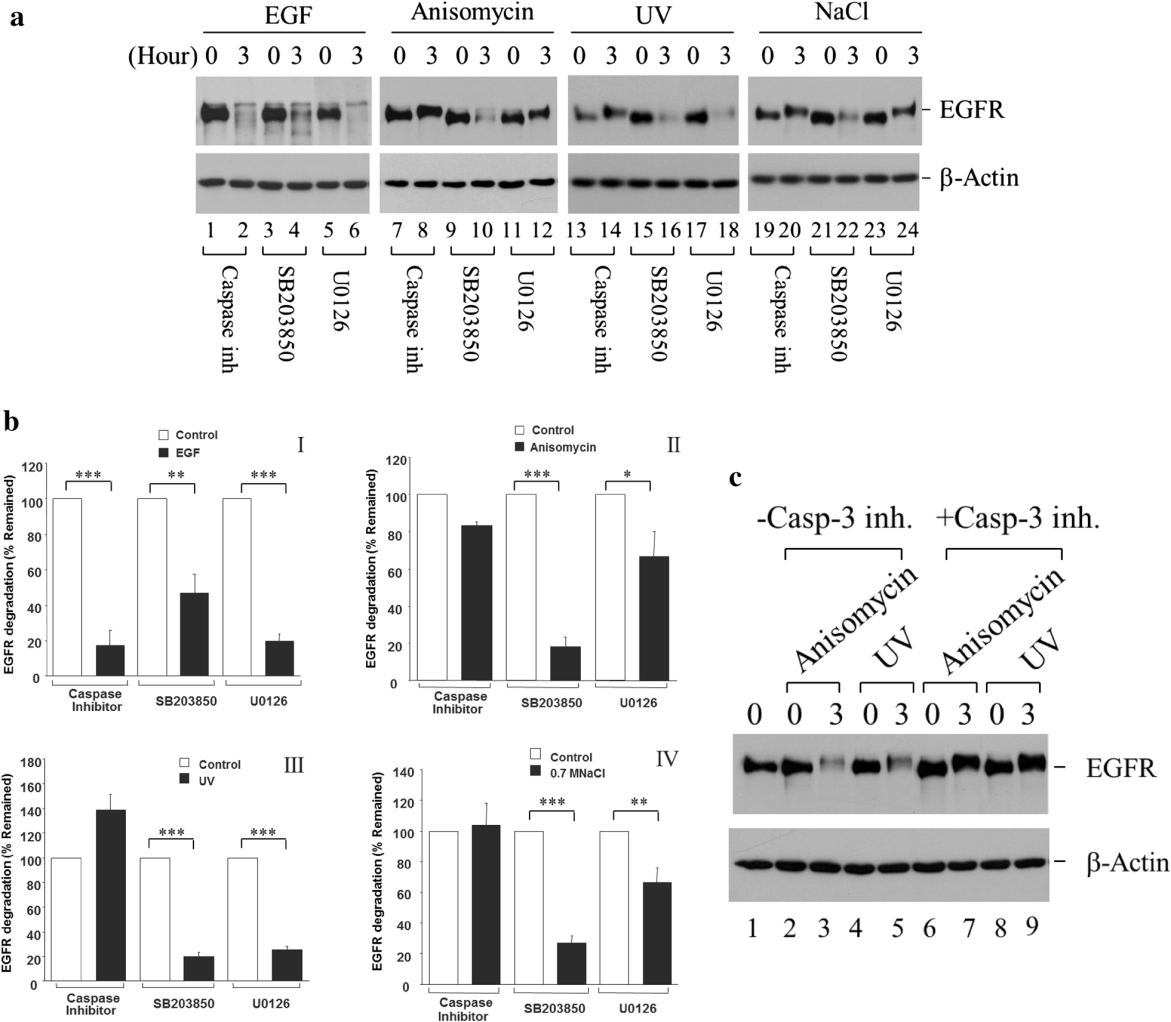
Caspase inhibitor blocks stress-induced degradation but not EGF (ligand)-induced degradation of EGFR. HeLa cells were cultured and serum-starved the same as described in Fig. 1. The caspase inhibitor mix (the caspase inhibitor mix, 2 μM each), Caspase-3 inhibitor (20 μM), p38 kinase inhibitor SB203850, or MEK1/2 inhibitor U0126 was added to the cells 30 min before the treatment of EGF, anisomycin, 0.7 M NaCl, or UV irradiation. The treatment of EGF and the stress conditions for inducing EGFR degradation was for 3 h. EGFR was immunoprecipitated with anti-EGFR(Mab528) and detected by immunoblotting with anti-EGFR (1005). **a** and **c** Western blot of EGFR (*the top panel*) and β-actin in whole cell lysates (*the bottom panel*) with immunoblotting. **b** Quantification of the immunoblotting data from three repetitions of experiments in (**a**). *p < 0.05, **p < 0.01, ***p < 0.001

Ligand-induced degradation of EGFR requires activation of EGFR because the E3 ubiquitin-ligase Cbl is recruited to active EGFR by binding to phosphotyrosine 1045 (pY1045) for ubiquitination of EGFR and Cbl-catalyzed ubiquitination is the sorting signal for transport to lysosomes for degradation. This mechanism does not apply to stress-induced degradation of EGFR because no activation of EGFR or Cbl-binding to EGFR is required for stress-induced degradation of EGFR. Previous studies demonstrated that stress-induced activation of p38 kinase or Caspase-3 plays an important role in anisomycin- and UV irradiation-induced degradation of EGFR [22, 25]. However, the molecular mechanism underlying stress-induced degradation of EGFR has not been fully understood. To elucidate the molecular mechanism underlying stress-induced degradation of EGFR, we first examined the effect of inhibitors of caspases, p38 kinase (SB203850) or MEK1/2 (U0126) on stress-induced degradation of EGFR. As shown in Fig. 2a, b, caspase inhibitor mix blocked all stress-induced degradation of EGFR (lanes 7, 8, 13, 14, 19 and 20, Fig. 2a; panels II, III, and IV, Fig. 2b), but did not affect ligand (EGF)-induced degradation of EGFR (lanes 1 and 2, Fig. 2a; panel I, Fig. 2b), indicating that all stress-induced degradation of EGFR is mediated by caspases, while ligand (EGF)-induced degradation of EGFR is not dependent on caspase activity. To our surprise, p38 inhibitor SB203850 had a minor inhibitory effect (about 30 %) on ligand (EGF)-induced degradation of EGFR (lanes 3 and 4, Fig. 2a; panel I, Fig. 2b), and no effect on stress-induced degradation of EGFR (lanes 9, 10, 15, 16, 21, and 22, Fig. 2a; panels II, III, and IV, Fig. 2b), suggesting that p38 kinase activity is not required for stress-induced degradation of EGFR. Interestingly, the MEK1/2 inhibitor U0126 had a partial inhibitory effect (50 %) on anisomycin- and 0.7 M NaCl-induced degradation of EGFR (lanes 11, 12, 23, and 24, Fig. 2a; panels II and IV, Fig. 2b), without affecting ligand (EGF)-induced and UV irradiation-induced degradation of EGFR (lanes 5, 6, 17, and 18, Fig. 2a; panels I and III, Fig. 2b). Currently, the mechanism underlying inhibitory effect of the MEK1/2 inhibitor U0126 on the stress-induced EGFR degradation is unknown.

It has been reported that EGFR has caspase cleavage sites and is a substrate of Caspase-3 [25]. To further confirm that Caspase-3 is crucial for degradation of EGFR, we determined effect of an inhibitor of Caspase-3 on stress-induced degradation of EGFR. As shown in Fig. 2c, the Caspase-3 inhibitor specifically inhibited the degradation induced by anisomycin and UV-irradiation, indicating that Caspase-3 is the caspase involved in stress-induced degradation of EGFR.

Taken together, stress-activated Caspase-3 activity is the cause for stress-induced degradation of EGFR, while stress-activated p38 kinase activity is not required for stress-induced degradation of EGFR.

### Stress-activated p38 kinase activity but not Caspase-3 activity is required for stress-induced endocytosis of EGFR

**Fig. 3:**
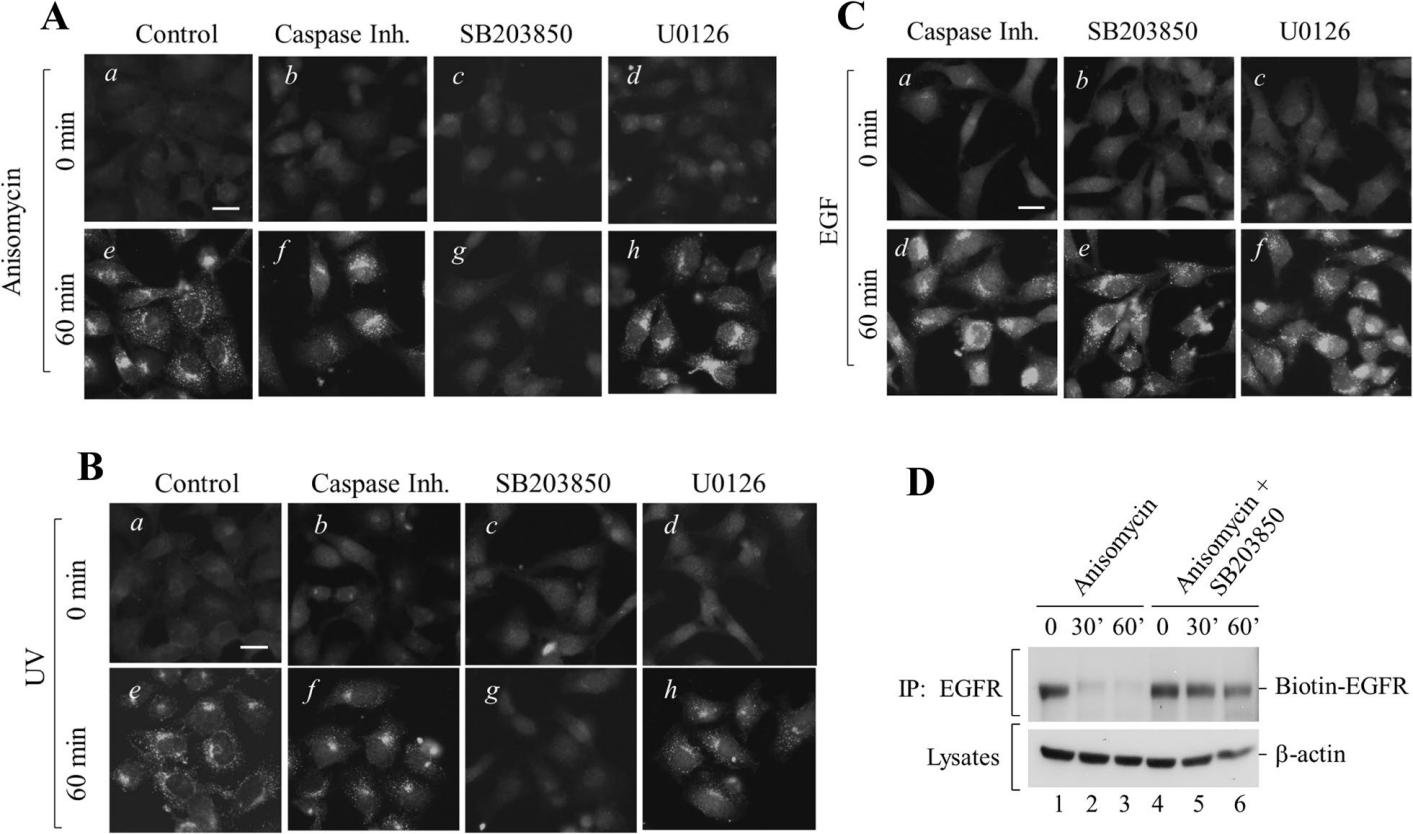
p38 kinase activity is required for stress-induced endocytosis of EGFR. **A**–**C** HeLa cells were cultured on glass cover slip-bottomed dishes (Matek) and serum-starved in DMEM plus 1 % FBS for 12 h. The Caspase-3 inhibitor, p38 kinase inhibitor SB203850 or MEK inhibitor U0126 was added into the medium 30 min before the treatment of EGF, anisomycin or UV irradiation. Then the cells were fixed and immunostained with anti-EGFR(1005) as described in “Methods” section. *Bar* 20 μm. **D** HeLa cells were biotin-labeled after treatment of anisomycin. The biotin-labeled EGFR was immunoprecipitated by anti-EGFR(Mab528) and detected by immunoblotting with anti-biotin (*the top panel*). The relative amount of lysates used for immunoprecipitation was controlled by immunoblotting of β-actin in the lysates (*the bottom panel*)

It has been shown that stress-induced endocytosis of EGFR is independent of ligand stimulation and impaired by inhibition of the p38 kinase activity [22]. To define the role of p38 kinase in stress-induced degradation of EGFR, we examined effects of inhibitors of Caspase-3, p38 and MEK1/2 kinases on stress-induced endocytosis assayed by immunofluorescent staining with anti-EGFR. As shown in Fig. 3A, B, after 60 min treatment of anisomycin or UV-irradiation, EGFR was endocytosed (panels a and e). The treatment of inhibitors of Caspase-3 and MEK1/2 kinases on the cells did not block the stress-induced endocytosis of EGFR (panels b, d, f and h, Fig. 3A, B). However, SB203850, the inhibitor of p38 kinase, completely diminished the stress-induced endocytosis of EGFR (panels c and g, Fig. 3A, B). As a control, we examined the effect of the inhibitors on ligand-induced endocytosis of EGFR, as shown in Fig. 3C. Consistent with the effect on ligand-induced degradation of EGFR, the inhibitors did not affect the ligand-induced endocytosis of EGFR, suggesting that ligand-induced endocytosis utilizes a different mechanism from that of stress-induced endocytosis.

To confirm that p38 kinase inhibitor blocks stress-induced endocytosis of EGFR, we also employed an alternative biochemical approach in addition to the immunofluorecent staining. We labeled cell surface EGFR with biotin at different time points of anisomycin treatment in presence or absence of the p38 kinase inhibitor SB203850. The biotin-labeled EGFR was then immunoprecipitated with anti-EGFR (Mab528) and detected by immunoblotting with anti-EGFR (1005) (Santa Cruz). As shown in Fig. 3D, endocytosis of EGFR reached to more than 90 % of completion upon 30 min treatment (lane 2, upper panel) and 100 % of completion upon 60 min treatment of anisomycin (lane 3, upper panel). Addition of the p38 kinase inhibitor SB203850 to the anisomycin treatment blocked more than 90 % of anisomycin-induced endocytosis of EGFR (lanes 4–6, upper panel, Fig. 3D), confirming that stress-activated p38 kinase activity is indeed required for stress-induced endocytosis of EGFR.

Taken together, these data indicate that stress-activated p38 kinase activity is required for stress-induced endocytosis of EGFR, while Caspase-3 and MEK1/2 kinase activity are not involved in stress-induced endocytosis. In addition, these data have demonstrated that stress-induced endocytosis and degradation of EGFR are two independent cellular processes, which is different from that of ligand-induced endocytosis and degradation.

### Proteasomal activity is required for stress-induced degradation but not endocytosis of EGFR

**Fig. 4:**
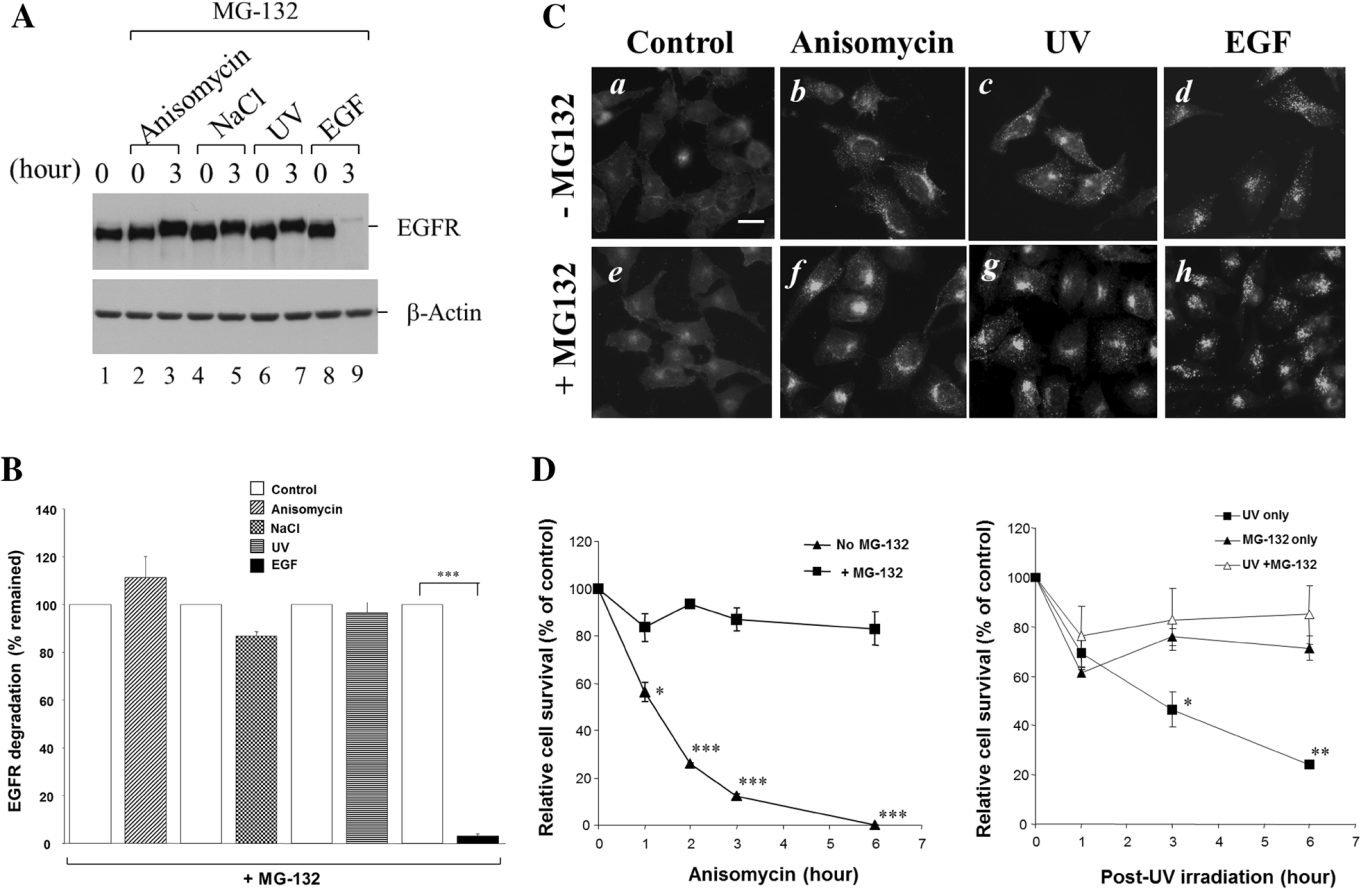
Proteasomal activity is required for stress-induced degradation of EGFR and cell death, but not endocytosis of EGFR. HeLa cells were cultured and serum-starved the same as described in Fig. 1. The proteasome inhibitor MG-132 (10 μM) was added to the cells 30 min prior to the treatment of EGF, anisomycin, UV irradiation or 0.7 M NaCl. **A** The cells were lysed and EGFR was immunoprecipitated and detected by immunoblotting with anti-EGFR(1005) (*the top panel*). **B** The immunoblotting data of EGFR from three repetitions of experiments was quantified by Kodak EDAS290 image system. ***p < 0.001. **C** Examination of effect of MG-132 on stress-induced endocytosis of EGFR by immunofluorescent staining of EGFR. The experimental procedures were the same as described in Fig. 3A–C *Bar* 20 μm. **D** MG-132 protects stress-induced cell death. The cells were incubated in MG-132 (10 μM)-contained medium 30 min prior to the treatment of anisomycin or UV irradiation. The viable cell numbers were counted under a microscope with a hemacytometer. The relative cell survival was plotted as percentage of the control cell numbers. In statistical analysis, the samples treated with MG-132 alone are used as the control data set. *p < 0.05, **p < 0.01, ***p < 0.001

Previous studies have shown that the proteasome inhibitor MG-132 completely blocked anisomycin-induced degradation of EGFR and proposed that proteasomes may execute the anisomycin-induced degradation of EGFR [22]. To examine the role of proteasome in stress-induced endocytosis and degradation, we treated HeLa cells with MG-132 prior to stress treatments. As shown in Fig. 4A, B, MG-132 completely blocked anisomycin-, 0.7 M NaCl- and UV irradiation-induced degradation of EGFR (lanes 2–7, Fig. 4A, B), but not ligand (EGF)-induced degradation of EGFR (lanes 8 and 9; Fig. 4A, B). This data suggest that the proteasome activity is required for stress-induced degradation of EGFR.

To determine if MG-132 affects stress-induced endocytosis of EGFR, we performed immunofluorescent staining with anti-EGFR under stress conditions in presence or absence of MG-132. As shown Fig. 4C, MG-132 did not significantly affect the anisomycin- 0.7 M NaCl- and UV-irradiation-induced endocytosis of EGFR, suggesting that proteasomal activity is not essential for stress-induced endocytosis.

Because ubiquitination of EGFR has not been observed upon stress treatments [22], it is unlikely that proteasomal activity directly mediates stress-induced degradation of EGFR. Furthermore, Caspase-3 activity is essential for stress-induced degradation of EGFR (Fig. 2). Thus, we speculate that proteasomal activity may be required for stress-induced activation of Caspase-3. In fact, we observed that MG-132 protected the cells from apoptosis induced by treatment of anisomycin or UV irradiation as evaluated by cell survival rate (Fig. 4D), strongly suggesting that proteasome activity is required for caspase-initiated apoptosis, as described by Sohn et al. [34]. However, further investigation is needed to clarify the role of proteasomal activity in stress-induced activation of caspases and degradation of EGFR.

### Stress-induced endocytosis of EGFR may coordinate with the stress-induced degradation

**Fig. 5:**
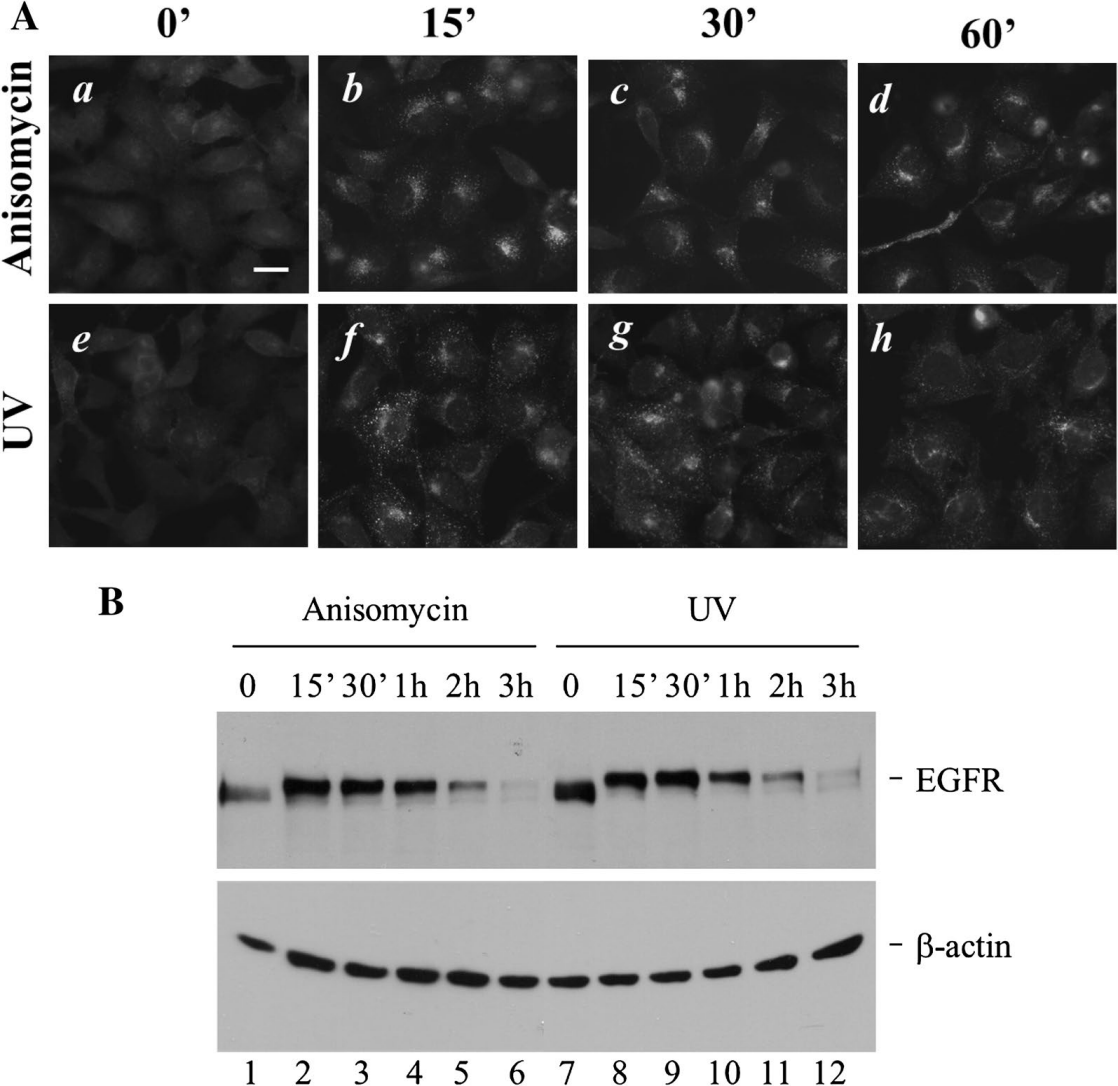
Stress-induced endocytosis is coordinated with the degradation of EGFR. The same batch of HeLa cells were used for examining the time scale of stress-induced endocytosis and degradation analyzed by immunofluorescent staining (**A**) or immunoblotting (**B**) with anti-EGFR(1005). The treatment of anisomycin or UV irradiation in samples for examination of EGFR endocytosis (immunofluorescent staining) was performed in parallel with that for immunofluorescent staining. The experimental procedures were the same as described in Figs. 1 and 2. *Bar* 20 μm

Although stress-induced endocytosis and degradation are two independent events, they may coordinate each other in cells to down-regulate EGFR and eliminate EGFR signaling under stress conditions. To examine the coordination between the endocytosis and the degradation, we performed the stress-induced endocytosis assay by immunofluorescent staining of EGFR in parallel with the stress-induced degradation assay by immunoblotting of EGFR and compared the progression in these two processes. As shown in Fig. 5A, endocytosis of EGFR induced by anisomycin or UV-irradiation reached to maximum level within 15 min treatment (panels b and f). At the same time point, the degradation of EGFR did not occur (lanes 2 and 8, Fig. 5B). The data in Fig. 5A, B indicate that stress-induced endocytosis of EGFR precedes stress-induced degradation of EGFR, implicating that stress-induced endocytosis and degradation could be two coordinated processes. The sequential order of stress-induced endocytosis and degradation of EGFR may be critical for cells to completely shut down EGFR signaling under stress conditions.

## Discussion

Our studies have shown that both chemical and physical stress conditions induce endocytosis and degradation of EGFR in cervical cancer HeLa cells (Fig. 1). By employing inhibitors of p38 kinase and Caspase-3, we demonstrated that activation of p38 kinase and Caspase-3 are two major biochemical processes in stress-induced endocytosis and degradation of EGFR (Fig. 2). Our data indicate that stress-induced endocytosis and degradation are two independent processes, since inhibition of p38 kinase impairs the endocytosis but not the degradation, while inhibition of Caspase-3 blocks the degradation but not the endocytosis (Figs. 2, 3).

In the ligand-induced degradation, EGFR is first internalized then transported to lysosomes for degradation, thus endocytosis is required for the degradation. In the stress-induced degradation, EGFR is directly cleaved by stress-activated Caspase-3, thus endocytosis is not required for the degradation. We observed that upon treatment with UV irradiation or anisomycin quickly induced endocytosis of EGFR much earlier than the degradation (Fig. 5), suggesting that the endocytosis might coordinate with the degradation. We speculate that stress-induced endocytosis of EGFR is critical for completion of down-regulation of cell surface EGFR signaling. Because Caspase-3 cleavage sites in EGFR are localized in the regulatory region of cytoplasmic portion [25], the Caspase-3-cleaved (degraded) EGFR is still able to bind to its ligands and partially transduce signals mediated by the kinase domain if it is retained on plasma membranes. The stress-induced endocytosis enables EGFR quickly to separate from its extracellular ligands thus to minimize the EGFR signaling. Therefore, stress-induced endocytosis might be necessary for completely shutting down the EGFR signaling. Consistent with our speculation, Zwang and Yarden [27] observed that p38 mediated stress-induced endocytosis of EGFR augmented cytotoxicity produced by chemotherapeutic agents in HeLa cells, suggesting that stress-induced endocytosis of EGFR is important for down-regulating EGFR-mediated anti-apoptotic signaling in the cervical cancer cells.

However, a recent research article showed that stress-induced endocytosis is required for endosomal EGFR activation and defect in the endocytosis enhances the UV irradiation- or cisplatin-induced apoptosis in HeLa cells [29], suggesting that the stress-induced endocytosis is important for EGFR to generate endosomal anti-apoptotic signaling. The role of the stress-induced endocytosis defined in this report is inconsistent with the report from Zwang and Yarden [27]. To clarify the discrepancy on the role of stress-induced endocytosis of EGFR in chemotherapy-induced cancer cell apoptosis, further studies are needed.

We observed that the MEK1/2 inhibitor U0126 had a significant inhibitory effect on anisomycin- or 0.7 M NaCl- induced degradation of EGFR (Fig. 2a, b), suggesting that MEK1/2 and Erk activation might be involved in stress-induced activation of Caspase-3 for degrading EGFR. There are several lines of evidence showing that activation of Erk is involved in stress-induced apoptosis [35, 36]. Our results are consistent with the observation that Erk activation is required for chemical stress (cisplatin treatment)-induced apoptosis but not for UV-induced apoptosis [37]. However, we do not know why Erk plays a role in chemical stress-induced apoptosis but not in UV-induced apoptosis. One possibility is that Erk is not involved in ROS-mediated stress signaling since PD98059 did not have an effect on oxidant-induced apoptosis [37]. Further investigation is required for understanding exact roles of MEK1/2 and Erk1/2 in regulation of stress signaling.

In addition, we observed that the proteasomal inhibitor MG-132 impaired stress-induced degradation, but not endocytosis of EGFR (Fig. 4), suggesting that proteasomal activity might be involved in regulation of the stress-induced degradation process. It has been reported that proteasomes down-regulate a number of ubiquitinated anti-apoptotic proteins [38]. The effect of MG-132 on protection of stress-induced degradation of EGFR may result from stabilization of ubiquitinated anti-apoptotic proteins. It is interesting to look into the role of ubiquitination in regulation of stress-induced degradation of EGFR in future studies.

How p38 kinase activation induces endocytosis of EGFR is still not fully understood. It has been observed that activation of p38 kinase facilitates ligand-induced degradation of EGFR in COS7 cells [39]. Inhibiting p38 kinase by the kinase inhibitor or knockdown by RNAi causes significant reduction of tyrosine phosphorylation at Y1045 of EGFR, which is required for EGFR binding to the E3 ubiquitin ligase Cbl, thus results in defect in ubiquitination and degradation of EGFR [39]. However, p38 kinase activity did not affect ligand-induced internalization of EGFR [39], which is consistent with our results shown in Fig. 3C. We observed only a partial inhibition of ligand-induced degradation of EGFR by suppression of p38 kinase activity with SB203850 (Fig. 2b-I). It has been observed that stress conditions, such as treatment with cisplatin, anisomycin or UV irradiation, induce phosphorylation of EGFR through activation of p38 [27]. This p38 mediated phosphorylation is required for stress-induced internalization of cell surface EGFR [40, 41]. Previous studies have demonstrated that the stress-induced endocytosis of EGFR was mediated by clathrin-coated vesicles [26–28]. The phosphorylation of EGFR by activated p38 kinase upon stress treatment might function in recruiting adaptor protein AP2 for assembly of clathrin-mediated endocytic complex of EGFR. However, the exact role of p38-mediated phosphorylation of EGFR in stress-induced endocytosis of EGFR is still not known and needs to be defined by further investigation.

## Conclusions

Stress conditions induced endocytosis and degradation of EGFR independent of ligand stimulation in cervical cancer HeLa cells. Unlike ligand-induced endocytosis and degradation that are consequential events, the stress-induced endocytosis and degradation are two independent events. Stress-induced endocytosis of EGFR is dependent on p38 kinase and the degradation is catalyzed by Caspase-3. In addition, proteasomal activity is also required for stress-induced degradation, but not endocytosis, of EGFR. We propose that the cancer cells utilize a double-secure way to down-regulate cell surface EGFR in response to stress conditions.

## Methods

### Materials

EGF was purchased from Invitrogen (Gibco); SB203850, U0126 and MG-132 were purchased from CalBiochem; Caspase inhibitor mix and Caspase-3 inhibitor were purchased from Biovision; anisomycin and puromycin were purchased from Sigma. Anti-EGFR antibody (1005) was purchased from Santa Cruz Biotechnology; anti-EGFR Mab528 was obtained from the culture medium of the hybridoma cell line 528 purchased from ATCC. Anti-β-Actin was purchased from Sigma. The biotin-labeling kit was purchased from Pierece.

### Cell culture and treatments

The cervical cancer HeLa cells were cultured in DMEM plus 10 % FBS with 5 % CO2. The cells were starved in serum-free medium overnight before the treatment. The time course of the treatments, including EGF (100 ng/ml), anisomycin (60 μM), puromycin (5 μg/ml) and NaCl (0.7 M), was done in a reverse way, i.e., the longest treatment was done first and the shortest treatment was done last, so all the treated cells were harvested at the same time. For inhibition of Caspase-3, p38 kinase and MEK1/2 kinase activity, the inhibitors were added 30 min before the treatments of EGF, anisomycin, NaCl, or UV irradiation. For UV irradiation, the culture medium was removed and the cells on culture dishes were directly exposed to UV irradiation at about 8 J/cm^2^ for 2 min, subsequently cultured in replenished culture medium for indicated time (referred as UV treatment time).

### Immunoprecipitation and immunoblots

The cells were rinsed with PBS once and lysed with the lysis buffer (40 mM Hepes, pH 7.4, 100 mM NaCl, 1 % Triton X-100, 25 mM β-glycerophosphate, 1 mM sodium orthervanadate, 10 μg/ml leupeptin and 10 μg/ml aprotinin) (0.5 ml/60 mm dish) with rocking at 4 °C for 20 min. The cell lysates were collected into an eppendorf tube and cleared by centrifugation at 14,000 rpm for 4 min in a microfuge. The cleared lysates were transferred to a clean eppendorf tube and incubated with primary antibody on ice for 30 min, then protein A or protein G beads were added and the mixture is incubated at 4 °C for 2 h with rotation. The beads were washed with lysis buffer three times. The immunoprecipitation complexes were directly dissolved in SDS-PAGE sample buffer and separated by SDS-PAGE. The immunoblot was performed as instructed by ECL immunoblot kits (Amersham).

### Immunofluorescent staining

The cells were cultured in glass bottom microwell dishes (MatTek) to 50–80 % confluence. After the culture medium was removed, the cells were rinsed with PBS twice, fixed with 3.7 % paraformaldehyde at 25 °C for 10 min and permeablized with 0.2 % Triton X-100 in PBS at 25 °C for 10 min. Following washing with PBS, the cells were incubated with primary antibody at 25 °C for 60 min. Then the cells were washed with PBS three times and incubated with secondary antibody that was conjugated with a fluorescent dye at 25 °C for 60 min. Finally, the cells were washed with PBS three times (for 10 min each) and incubated in PBS. Immunofluorescence staining was visualized with a Nikon inverted fluorescent microscope.

### Biotin labeling in determination of stress-induced internalization of EGFR

HeLa cells were cultured in DMEM plus 10 % FBS to 90 % confluence, then serum-starved for 6 h, following by the treatment with anisomycin (60 μM) for indicated time. The p38 kinase inhibitor SB203850 was added to the cells 30 min prior to anisomycin treatment. For biotin labeling of cell surface EGFR, pre-cooled (4 °C) sulfo-NHS-SS-biotin (Pierce) (1.5 mg/ml) in DMEM was added to the cells immediately after anisomycin treatment, and incubated with the cells on ice for 30 min for labeling cell surface proteins. The cells were lysed with the lysis buffer and EGFR was immunoprecipitated with anti-EGFR (Mab528). The biotin-labeled EGFR was detected by immunoblotting with an anti-biotin antibody. The equality of amount of cell lysates used for immunoprecipitation among the samples was evaluated by the amount of β-actin in the lysates that was determined by immunoblotting with anti-β-actin antibody.

### Cell viability assay

Cells were plated to a 12-well plate incubating in 1 ml of media per well. Each treatment group was plated and counted in triplicate. At the indicated times for counting, the media was removed and 0.5 ml of trypsin/0.2 %EDTA added. Fifty microliter of 0.4 % Trypan Blue (MediaTech) added to clean eppendorf tube. The trypsinized cells were added to this same eppendorf tube. After 5 min, 10 μl of the suspended cells were added to a Bright-Line Hemacytomer (Hausser Scientific). Only live cells were counted. The relative cell survival was calculated and defined as a percentage of the control cell number.

### Statistical analysis

Student’s t test was used in statistical analysis of experimental data. The *p* value less than 0.05 is considered as statistical significance.
